# Care of undocumented-uninsured immigrants in a large urban dialysis unit

**DOI:** 10.1186/1471-2369-13-112

**Published:** 2012-09-19

**Authors:** Gil Chernin, Amir Gal-Oz, Idit F Schwartz, Moshe Shashar, Doron Schwartz, Talia Weinstein

**Affiliations:** 1 Nephrology Department, Tel-Aviv Sourasky Medical Center and Sackler Faculty of Medicine, Tel-Aviv University, Tel-Aviv, 64239, Israel

**Keywords:** Dialysis, ESRD, Undocumented, Uninsured, Immigrants

## Abstract

**Background:**

Medical, ethical and financial dilemmas may arise in treating undocumented-uninsured patients with end-stage renal disease (ESRD). Hereby we describe the 10-year experience of treating undocumented-uninsured ESRD patients in a large public dialysis-unit.

**Methods:**

We evaluated the medical files of all the chronic dialysis patients treated at the Tel-Aviv Medical Center between the years 2000–2010. Data for all immigrant patients without documentation and medical insurance were obtained. Clinical data were compared with an age-matched cohort of 77 insured dialysis patients.

**Results:**

Fifteen undocumented-uninsured patients were treated with chronic scheduled dialysis therapy for a mean length of 2.3 years and a total of 4953 hemodialysis sessions, despite lack of reimbursement. All undocumented-uninsured patients presented initially with symptoms attributed to uremia and with stage 5 chronic kidney disease (CKD). In comparison, in the age-matched cohort, only 6 patients (8%) were initially evaluated by a nephrologist at stage 5 CKD. Levels of hemoglobin (8.5 ± 1.7 *versus* 10.8 ± 1.6 g/dL; p < 0.0001) and albumin (33.8 ± 4.8 *versus* 37.7 ± 3.9 g/L; p < 0.001) were lower in the undocumented-uninsured dialysis patients compared with the age-matched insured patients at initiation of hemodialysis therapy. These significant changes were persistent throughout the treatment period. Hemodialysis was performed in all the undocumented-uninsured patients *via* tunneled cuffed catheters (TCC) without higher rates of TCC-associated infections. The rate of skipped hemodialysis sessions was similar in the undocumented-uninsured and age-matched insured cohorts.

**Conclusions:**

Undocumented-uninsured dialysis patients presented initially in the advanced stages of CKD with lower levels of hemoglobin and worse nutritional status in comparison with age-matched insured patients. The type of vascular access for hemodialysis was less than optimal with regards to current guidelines. There is a need for the national and international nephrology communities to establish a policy concerning the treatment of undocumented-uninsured patients with CKD.

## Background

Immigrants and refugees are a vulnerable population in all countries. It was estimated in 2005 that global migration has produced some 17 million “people of concern” (*i.e.* immigrants and refugees) worldwide and around 20–30 million irregular migrants (undocumented/illegal migrants) [1,2]. There is a great potential for health problems in this population as many have left countries that have limited health-care resources. Further compounding their plight is the fact that many are not granted public health insurance in the countries that receive them and they cannot afford to pay for health care expenses [1,2].

The initial wave of labor migration to Israel is dated to 1993, when employers where first granted a state permission to employ documented immigrants. Over less than a decade, individuals from over 90 countries immigrated to Israel with intention to work [3,4]. The majority of documented migratory workers came from Asia (mostly the Philippines) and Eastern Europe [5]. A report by the Israeli Parliament from 2009 estimated that 118,000 undocumented individuals live in Israel, the majority of whom reside in the Tel-Aviv metropolitan [6]. Most of the undocumented-immigrants in Israel were initially documented with permission to work. Once their staying permit had expired they chose to stay and continue working with the risk of deportation [3,6]. Employing undocumented-immigrants in Israel is illegal and is a subject for financial fine or for criminal charges.

Healthcare for Israeli citizens is universal and participation in a medical insurance plan is compulsory. For more than decade, it is also mandatory for documented- immigrant workers to have a full medical insurance plan, provided by their employer. In contrast, since 2003, it is impossible, by Israeli law, for undocumented-immigrants to purchase a medical insurance plan that is identical to the plan provided for citizens and documented-immigrants [3]. Nevertheless, certain medical services are provided by the state for undocumented-immigrants free of charge. It includes: emergency admission to hospitals, treatment of infants, toddlers and children at schools, tuberculosis therapy, and anti-viral therapy during pregnancy for women that carry the human-immunodeficiency virus (HIV) [3]. A special clinic designated for the care of undocumented-uninsured immigrants is located in Tel Aviv. It is operated by the Israeli ’Physicians for Human Rights’ organization and the medical staff of the clinic consists of volunteers [3,4].

Recent reports from industrialized countries have highlighted the medical as well as the ethical and financial dilemmas that arise while treating undocumented-uninsured end stage renal disease (ESRD) patients with the need for renal replacement therapy (RRT) [7-16]. Currently, there are no specific legal guidelines in Israel regarding chronic dialysis treatment for undocumented-uninsured patients with ESRD. This lack of policy creates a challenge for the treating nephrologists and hospitals. A primary consideration is to decide about the nature of dialysis therapy as either a chronic scheduled regimen of therapy or as an “emergent” therapy for overt symptoms attributed to ESRD [10,16,17].

Hereby, we describe a 10-year experience of treating fifteen undocumented-uninsured immigrants with ESRD in a large urban dialysis unit in Tel-Aviv. Our hypothesis was that undocumented-uninsured chronic kidney disease (CKD) patients have a late referral to nephrology consultants which may have adverse effects on clinical outcomes. We also examined whether the delay in CKD care, as well as other factors (e.g. lack of routine medications, possible no-show to dialysis) might be associated with different clinical outcomes during dialysis therapy. For these purposes, we compared the undocumented-uninsured patients with a cohort of age-matched insured patients on chronic dialysis therapy.

## Methods

### Patients and data recruitment

Approval of the study was granted by the Ethical Committee of the Tel-Aviv Medical Center. Medical records were obtained for patients that were treated with dialysis therapy from January 1^st^ 2000 to December 31^st^ 2010. From these data we evaluated only the chronic maintenance dialysis patients (*i.e.* more than 3 months of dialysis therapy) without a permit of stay and medical insurance (undocumented-uninsured cohort). The data on the legal status of the immigrant patients in Israel (*i.e.* lack of documentation) were acquired from the periodical reports of the social workers in the dialysis unit. To avoid technical errors or late payments, we then rechecked these data for each patient with the accounting department of the Tel-Aviv Medical Center. We evaluated the demographic, social and medical parameters and compared the clinical data of the undocumented-uninsured patients with a cohort of 77 age-matched insured dialysis patients in our hemodialysis unit. Clinical parameters that were compared included: CKD stage of initial nephrology evaluation, etiology of CKD, occurrence of viral infections [hepatitis B (HBV), hepatitis C (HCV) and human immunodeficiency virus (HIV)]; the type of hemodialysis vascular access, rate of tunneled cuffed catheters (TCC) associated infections, rate of skipped dialysis sessions (“no-show”) and levels of hemoglobin, albumin, parathyroid hormone (PTH) and C-reactive protein (CRP). CKD stages were defined according to the estimated glomerular filtration rate (eGFR) [18]. eGFR was calculated with the use of the four-variable MDRD equations [19]. TCC-associated infections were defined as: (*i*) A blood culture drawn from a catheter that has a ≥3-fold greater colony count of microbiologic isolates than those drawn from a peripheral vein (definite bacteremia) or (*ii*) Positive blood cultures obtained from a catheter and/or a peripheral vein in a symptomatic patient when there is no clinical evidence for an alternative source of infection [20].

### Statistical analysis

Two-sample Student *t*-test was used to compare the 15 undocumented-uninsured dialysis patients and the cohort of 77 age-matched documented-insured dialysis patients. Variables that were compared included: age at initiation of RRT, days of RRT, days in hemodialysis with TCC, TCC-associated infections per 1000 days of dialysis treatment, hemoglobin at initiation of RRT, hemoglobin during RRT follow-up, albumin at initiation of RRT, albumin during RRT follow-up, PTH at initiation of RRT, PTH during RRT follow-up, CRP during RRT follow-up, skipped HD sessions (“no-show”). Statistical analysis was performed by SAS for Windows Version 9.2.

## Results

### Patient characteristics

Twenty-eight undocumented-uninsured patients were treated in our dialysis unit with RRT between the years 2000–2010. Thirteen undocumented-uninsured patients were treated with hemodialysis for less than three months. Of these 13 patients, eight patients had a reversible acute kidney injury or intoxication without the need for chronic dialysis therapy and four patients died in hospital. One patient chose to discharge himself without returning to dialysis treatment.

Fifteen undocumented-uninsured patients were treated with chronic maintenance dialysis therapy (*i.e.* more than 3 months of dialysis therapy). These 15 undocumented-uninsured patients are the cohort described below. Dialysis therapy for these patients was initiated during hospitalization. Once it was concluded by the treating nephrologist that they suffered from ESRD and needed to continue dialysis therapy, they were discharged and scheduled for thrice weekly dialysis sessions. The decision to provide chronic dialysis was authorized by the hospital administration, despite the lack of medical insurance, based on humanitarian grounds.

Baseline clinical characteristics are summarized in Table 1. The geographical origin of 15 undocumented-uninsured patients was in general from 3 regions: West Africa, the Philippines and Eastern Europe (Table 1). Fourteen patients entered the state of Israel more than one year before initiating RRT. Twelve patients had expired legal permit to stay in Israel with expired medical insurance. All 15 patients worked regularly during the treatment period: in construction (n = 4; all males), nursing (n = 1) or housekeeping (n = 10).

**Table 1:** Baseline characteristics of undocumented-uninsured patients in comparison with insured patients on chronic dialysis

**Clinical Characteristics**	**Undocumented-Uninsured (n = 15)**	**Age-matched Insured (n = 77)**	**Insured- General Population (n = 223)**
**Age (Years** ± **SD)**	41.1 ± 11.6	43.2 ± 10.2	67.6 + 14.7
**Sex (male/female)**	9/6	50/27	137/86
**Origin and status**			
**Undocumented-uninsured**			
West Africa	8	-	-
Philippines	5	-	-
Eastern Europe	2	-	-
**Insured**			
Israel Jewish	-	73	216
Israel-Arabs	-	3	6
Non Israeli	-	1	1
Working status (%)*			
Full-Time work	15 (100%)	12 (16%)	16 (7%)
Part-Time work	-	3 (4%)	5 (2%)
Not working	-	62 (80%)	202 (91%)
**CKD stage of initial nephrology evaluation (%)**			
Stage 1	0 (0%)	18 (23%)	25 (11%)
Stage 2	0 (0%)	17 (22%)	20 (9%)
Stage 3	0 (0%)	21 (27%)	65 (29%)
Stage 4	0 (0%)	15 (20%)	79 (35%)
Stage 5	15 (100%)	6 (8%)	34 (16%)
**CKD etiology (%)**			
DN	0 (0%)	20 (26%)	47 (21%)
ADPKD	0 (0%)	5 (7%)	9 (4%)
Glomerulonephritis	0 (0%)	11 (14%)	21 (9%)
Other	1 (7%)	21 (27%)	55 (25%)
Unknown	14 (93%)	20 (26%)	91 (41%)
**Viral infection (%)**			
HBV	1 (7%)	5 (6%)	6 (3%)
HCV	2 (13%)	5 (6%)	14 (6%)
HIV	1 (7%)	1 (1%)	2 (1%)

Abbreviations: *ADPKD,* autosomal dominant polycystic kidney disease, *CKD*, chronic kidney disease; *DN*, diabetic nephropathy; *HBV*, hepatitis B virus infection; *HCV*, hepatitis C virus infection; *HIV*, human immunodeficiency virus infection; *SD*, standard deviation; * Working-status as reported by the patients.

The mean length of dialysis therapy was 2.3 years (range 0.4-9.1 years, median 1.5 years). Overall, the undocumented-uninsured patients had cumulative 410 months of dialysis therapy for a total of 4953 hemodialysis sessions. One patient was treated for 7.3 years on hemodialysis and another 1.8 years on peritoneal dialysis. The cost of care for the 15 patients was estimated to be 8,141,000 Israeli New Shekels (INS). Two of the 15 patients died during the treatment period and another six patients returned to their country of origin. Personal or familial reasons were given as a cause of departure by four of the six patients that returned to their country of origin. One patient obtained legal permit of stay after 1.7 years of hemodialysis therapy and for the purpose of this report we included only the treatment period without legal documentation and medical insurance.

### CKD characteristics

#### Referral to nephrology evaluation

The CKD stage, at which the ESRD patients were first evaluated by a nephrologist, is shown in Table 1. All 15 undocumented-uninsured patients presented initially with symptoms attributed to uremia or volume overload and with eGFR lower than 15 mL/min/1.73 m^2^. Thus, all were initially evaluated by a nephrologist in CKD stage 5. In comparison, in the age-matched control cohort of 77 insured patients, only six patients (8%) were first evaluated by a nephrologist at CKD stage 5.

#### CKD etiology

A kidney biopsy was performed in five of the 15 undocumented-uninsured patients in order to determine if their renal disease was treatable. Global sclerosis without a definite diagnosis of the underlying renal disease was found in four patients and in another patient a diagnosis was made of multiple myeloma with renal AL amyloidosis. Kidney biopsy was not performed in the other ten patients due to: (*i*) Small scarred kidneys on renal imaging (n = 9); (*ii*) Patient’s decline of kidney biopsy (n = 1). In comparison, the underlying kidney disease was apparent in 57 of the 77 patients (74%) in the age-matched insured cohort (Table 1).

### Clinical outcome

#### Dialysis mode and vascular access

All 15 undocumented-uninsured dialysis patients were treated with hemodialysis performed *via* TCC. One patient was initially on hemodialysis for 7.3 years of therapy before a change to peritoneal dialysis was necessary due to a technical failure to insert a novel TCC. In comparison, in the age-matched control group of 77 insured hemodialysis patients, seventeen were treated *via* TCC (22%), 58 *via* A-V fistulas (75%) and another two patients *via* A-V grafts (3%). Surgery for A-V access creation was offered to four of the 15 undocumented-uninsured patients but was declined due to patient’s concerns regarding the need for hospitalization and the potential loss of physical labor capabilities with the operated limb.

The rate of catheter-related infections was 2.4 ± 1.8 episodes/1000 TCC days in the undocumented-uninsured dialysis patients compared with 2.0 ± 1.5 episodes/1000 TCC days in the 17 insured patients treated *via* TCC (P = 0.16). The KT/V measures during follow-up were 1.31 ± 0.16 in the undocumented-uninsured dialysis patients and 1.31 ± 0.25 in the insured patients (P = 0.2).

### Hemoglobin, albumin and PTH levels

#### At initiation of RRT

Advanced CKD can cause anemia, secondary hyperparathyroidism and a decline in nutritional status. We chose to evaluate levels of hemoglobin, albumin and PTH as parameters that define adequacy of CKD management and also predict mortality [21,22].

**Figure 1:**
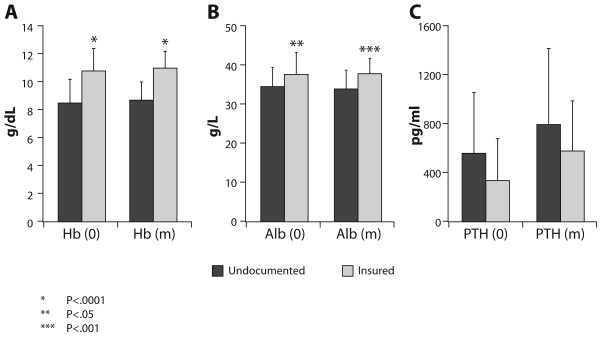
**Comparison of hemoglobin, albumin and PTH levels between 15 undocumented-uninsured dialysis patients and 77 age-matched insured patients. A**. Hemoglobin levels at initiation of renal replacement therapy [Hb (0)] and during dialysis treatment period [Hb (m)]. **B**. Albumin levels at initiation of renal replacement therapy [Alb (0)] and during dialysis treatment period [Alb (m)]. **C**. PTH levels at initiation of renal replacement therapy [PTH (0)] and during dialysis treatment period [PTH (m)].

The average hemoglobin levels at initiation of RRT [Hb(0)] were significantly lower for the undocumented-uninsured group in comparison with the age-matched group of insured patients (8.5 + 1.7 g/dL *versus* 10.8 + 1.6 g/dL; P < 0.0001, Figure 1A).

The average serum albumin levels at initiation of RRT [(Alb(0)] were significantly lower for the undocumented-uninsured group in comparison with the age-matched group of insured patients (34.4 + 4.9 g/L *versus* 37.5 + 5.6 g/L; P < 0.05, Figure 1B).

The average PTH levels at initiation of RRT [(PTH(0)] were higher but without statistical significance for the undocumented-uninsured group in comparison with the age-matched group of insured patients (559.6 + 494.5 pg/ml *versus* 336.5 + 342.0 pg/ml; P = 0.1, Figure 1C).

#### During the dialysis treatment period

The average hemoglobin levels during the dialysis treatment period [(Hb(m)] were significantly lower for the undocumented-uninsured group in comparison with the age-matched group of insured patients (8.7 + 1.3 g/dL *versus* 11.0 + 1.2 g/dL; P < 0.0001, Figure 1A).

The average serum albumin levels during the dialysis treatment period [(Alb(m)] were significantly lower for the undocumented-uninsured group in comparison with the age-matched group of insured patients (33.8 + 4.8 g/L *versus* 37.7 + 3.9 g/L; P < 0.001, Figure 1B).

The average PTH levels during the dialysis treatment period [(PTH(m)] were higher but without statistical significance for the undocumented-uninsured group in comparison with the age-matched group of insured patients (793.0 + 620.2 pg/ml *versus* 577.4 + 409.2 pg/ml; P = 0.2, Figure 1C).

### CRP levels during dialysis treatment period

Routine CRP levels were obtained in eleven of the 15 undocumented-uninsured patients and in 74 of the 77 insured patients. The average CRP levels during the dialysis treatment period were similar for the undocumented-uninsured group in comparison with the age-matched group of insured patients (4.1 ± 12.5 mg/L *versus* 2.9 + 10.9 mg/L; P = 0.1, Table 2).

**Table 2:** Clinical characteristics of chronic dialysis therapy for undocumented-uninsured patients in comparison with age-matched insured patients

**Clinical Characteristics**	**Undocumented-Uninsured (n = 15)**	**Insured (n = 77)**
**Length of dialysis therapy (mean; median)**	2.3; 1.5	2.8; 2.0
**Vascular access (%)**		
A-V fistula	0 (0%)	58 (75%)
A-V graft	0 (0%)	2 (3%)
TCC	15 (100%)	17 (22%)
**TCC- associated infections (episodes per 1000 HD therapy)**	2.4 ± 1.8	2.0 ± 1.5
Initial hemoglobin levels (g/dL)*	8.5 + 1.7	10.8 ± 1.6
Initial Albumin levels (g/L)*	34.4 ± 4.9	37.5 ± 5.6
**Initial PTH levels (pg/ml)**	559.6 ± 494.5	336 ± 302
Hemoglobin levels during follow-up (g/dL)*	8.7 ± 1.3	11.0 ± 1.2
Albumin levels during follow-up (g/L)*	33.8 ± 4.8	37.7 ± 3.9
**PTH levels during follow-up (pg/ml)**	793.0 ± 620.2	577.4 ± 409.2
**CRP levels during follow-up (mg/L)**	4.1 ± 12.5 **	2.9 + 10.9
**KT/V**	1.31 ± 0.16	1.31 ± 0.25
**Skipped treatments (%)**	0.4 ± 1.3	0.1 ± 0.4

Abbreviations: *A-V*, arteriovenous; *CRP*, C-reactive protein; *HD*, hemodialysis; *PTH*, parathyroid hormone; *SD*, standard deviation; *TCC*, tunneled cuffed catheter; * P < 0.05; ** CRP levels obtained routinely for 11 undocumented-uninsured patients and 74 insured patients.

### Skipped dialysis treatments (“no-show”)

The rate of skipped hemodialysis sessions without prior notification (“no-show”) was similar for the undocumented-uninsured group in comparison with the age-matched group of insured patients (0.4 ± 1.3% *versus* 0.1 ± 0.4%; P = 0.4; Table 2).

## Discussion

In general, the population of undocumented-uninsured patients in this cohort was of young individuals that migrated to Israel in search of work and not due to their kidney disease. Importantly, all the undocumented-uninsured patients continued to work and were productive, after initiation of RRT. The patients migrated from three different geographical regions: the Philippines, West Africa and Eastern Europe. Not surprisingly, these regions are also the main origin of documented migratory-workers in Israel (5). However, it is impossible to determine whether any of these three ethnic groups of immigrants have disproportionally high rates of ESRD, since the demography of the undocumented-immigrants in the Tel-Aviv metropolitan is unknown.

The undocumented-uninsured patients were first diagnosed with renal disease in the advanced stages of CKD. On the other hand, the majority of age-matched insured patients were followed by a nephrologist from the early stages of CKD (Table 1). Early diagnosis and treatment of CKD may improve prognosis and lower morbidity and mortality [23,24]. Late referral of undocumented-immigrants to medical treatments with the risk of worse outcome and lower quality of care is not unique to CKD and ESRD. Previous reports have shown similar patterns of late referral of undocumented-immigrants in other fields of medicine such as antenatal care [25-27] and primary care [28,29]. Importantly, lack of health-services utilization was reported even when access to these services was not denied [27,29].

The undocumented-uninsured patients had equal weekly dialysis therapy as compared with the insured patients and the KT/V parameter reflects adequate dialysis dosage. Nevertheless, certain inequality was found in part because of the absence of medical insurance. Lack of reimbursement was the main cause that all the undocumented-uninsured patients were treated *via* TCC and not by the preferred A-V access [30]. Routine medications were not provided to the undocumented-uninsured patients because dialysis patients in Israel receive medications directly from their medical insurer and not from the dialysis unit. The lack of routine therapy with drugs such as erythropoiesis-stimulating agent or active vitamin D analogues may explain the persistently lower levels of hemoglobin and the trend towards higher PTH in the undocumented-uninsured patients as compared with the insured patients (Table 2, Figure 1). A possible option is to enroll undocumented-uninsured patients in ongoing clinical trials with free provision of medication by the sponsored-organization of the trials. However, one needs to remember that there are ethical dilemmas that recruiters for clinical trials need to address (Table 3) [31].

**Table 3:** Key issues in the management of undocumented-uninsured patients with CKD and ESRD in Israel

**Remarks**	**Issue**
**CKD therapy**	Need for early referral to nephrology consultant (the ’Physicians for Human Rights’ clinic in Tel-Aviv, other healthcare service?)
	Main aim: delaying CKD progression towards ESRD
**Dialysis therapy**	Scheduled weekly hemodialysis therapy *versus* “emergent” dialysis therapy
	Hemodialysis *versus* peritoneal dialysis
	Disparities of medical management (e.g. anemia, CKD mineral bone disease)
	Lowering costs (e.g. machine monitoring of KT/V instead of blood samples)
**Vascular access for hemodialysis**	Disparities due to lack of reimbursement
	Creation of A-V access *versus* the use of TCC
**Eligibility for clinical trials**	Translation for informed consent
	Fear of exploitation
	Continuity of therapy after termination of trial
**Public health**	Treatment of communicable diseases (e.g. tuberculosis)
**Renal transplantation**	Access to kidney transplantation
	Reimbursement of medications post-transplantation
**Legal**	Lack of documentation and possible deportation
	Fear of deportation as a potential cause for skipped therapy
	Avoiding return to country of origin if RRT is not accessible

Abbreviations: *A-V*, arteriovenous; *CKD*, chronic kidney disease; *ESRD*, end-stage renal disease; *RRT*, renal replacement therapy ; *TCC*, tunneled cuffed catheter.

Serum albumin levels did not increase in the cohort of undocumented-uninsured patients. Indeed, increased serum albumin over-time is associated with a better outcome in hemodialysis [21]. Inadequacy of dialysis [32] and persistent inflammation [33] are possible causes for malnutrition and hypoalbuminemia that were not found in the undocumented-uninsured patients. Hence, it is possible that other causes contributed to the persistency of hypoalbuminemia, such as lack of access to nutrition of good biological value.

Our study has limitations as it is a non-randomized, retrospective observation in a single dialysis unit. It does not take into account different trends over the years 2000–2010 in regards to anemia and CKD mineral bone disease management. Another limitation is that clinical data were compared between undocumented-uninsured patients and a cohort of insured-patients, of which only one patient was an immigrant. A comparison with a cohort of documented-insured immigrants was not done because of the small number patients in this group. Such a comparison could have tested the hypothesis that some differences that we observed were not primarily due to the status of legal stay but may in fact be related to ethnic or socio-economic status. A previous report, for example, suggested that the ethnic origin may influence hemoglobin levels in the CKD population [34].

Table 3 summarizes key medical, ethical and legal issues that, in our opinion, should be addressed regarding CKD management in the population of the undocumented-uninsured immigrants in Israel. In the case of ESRD, one need to decide about the nature of dialysis therapy and our experience suggests that scheduled weekly hemodialysis therapy may work well without excessive skipped hemodialysis sessions. Healthcare disparities such as the lack of reimbursement for the creation of A-V access are major ethical challenges for the caring nephrologists and the Israeli healthcare system. A proper balance is needed between the recommendations made by the medical profession and the obstacles raised by the legal status of these immigrants. It is plausible that we will witness a world-wide rise in the prevalence of undocumented-uninsured patients with ESRD. Therefore, we suggest that the national and international nephrology communities together with health-care organizations establish a consensus regarding the standard-of-care for undocumented-uninsured CKD patients.

## Conclusions

Undocumented-uninsured patients with ESRD had a late diagnosis of their CKD. Healthcare disparities were found with regard to the preferred hemodialysis vascular access and the lack of routine provision of medications. These findings suggest that new strategies for early surveillance and standard of care for undocumented-uninsured patients with CKD and ESRD are needed.

## Competing interests

The authors declare that they have no competing interests.

## Authors’ contributions

GC- participated in the design and draft of manuscript (the major contributor). AG- participated in the design of the study and performed the main statistical analysis. IFS- participated in the acquisition and analysing of clinical data. MS- participated of in the acquisition and analysing of clinical data. DS- participated in the design of the study, acquisition of data and draft of manuscript. TW- conceived of the study, and participated in its design and critically revisited the draft. All authors read and approved the final manuscript.

## Pre-publication history

The pre-publication history for this paper can be accessed here:

http://www.biomedcentral.com/1471-2369/13/112/prepub
